# Faecal occult blood testing in persons aged 50–74 years with established spinal cord injury: a prospective case series

**DOI:** 10.1038/s41394-025-00710-4

**Published:** 2025-05-09

**Authors:** Michael Yulong Wu, Carmen Tung, McCawley Clark-Dickson, Samuel Arthurs, Steffanie Nario, Ian D. Norton

**Affiliations:** 1 Department of Gastroenterology and Hepatology, Royal North Shore Hospital, St Leonards, Sydney, NSW Australia; 2 Northern Clinical School, University of Sydney, Sydney, NSW Australia; 3 Spinal Cord Injury Unit, Royal North Shore Hospital, St Leonards, Sydney, NSW Australia

**Keywords:** Colorectal cancer, Cancer screening, Diagnosis, Outcomes research

## Abstract

**Study design:**

Prospective Case Series.

**Objectives:**

To determine the incidence of positive faecal occult blood test (FOBT) in people with spinal cord injury (SCI). We hypothesised that people with SCI have a higher false positive FOBT rate when compared to the general population due to the high prevalence of complications of chronic constipation, and colonic and anorectal trauma from instrumentation.

**Setting:**

Hospital in Sydney, New South Wales, Australia.

**Methods:**

A prospective study was conducted at a quaternary hospital with a dedicated spinal injuries unit. Enrolled individuals had two FOBT samples sent. Persons outside the age criteria, or with active per-rectal or vaginal bleeding, anorectal disease, haematuria, recent SCI or colonoscopy, or who had completed FOBT as part of the National Bowel Cancer Screening Program were excluded.

**Results:**

A total of 20 people were included in the study with 50% testing positive on FOBT. Three persons agreed to proceed with colonoscopy. All persons who declined colonoscopy were due to perceived difficulty with procedure preparation. FOBTs were positive in 90% of those who reported rectal enema, digital simulation or manual evacuation as part of their bowel care.

**Conclusion:**

People with SCI have higher rates of positive FOBT compared to the general Australian population whilst follow-up colonoscopy rates were low. Compliance may be improved by bowel preparation protocols. High rates of positive FOBT in this population may be related to complications of constipation and bowel care. Our results suggest that FOBT is not an accurate screening tool in this population.

## Introduction

Colorectal cancer (CRC) is the 4th most commonly diagnosed cancer in Australia comprising 9.3% of all new cancer diagnoses [1]. CRC is associated with significant morbidity and mortality, responsible for an estimated 10% of all cancer-related deaths in Australia [2]. In 2006, the Australian Government established the National Bowel Cancer Screening Program (NBCSP) to reduce the morbidity and mortality of CRC through the early detection of malignancy. The program involves a biennial faecal occult blood test (FOBT) for all Australians aged 50–−74 years of age and those who test positive are recommended to undergo further investigation with colonoscopy. FOBT has a reported sensitivity of 16–64% and specificity of 90.6% in the detection of advanced adenomas, increasing to a sensitivity of 52–92% and specificity of more than 90% in the detection of CRC [3–6]. CRC screening programs have been shown to reduce mortality and are a cost-effective intervention to reduce CRC incidence and mortality [7].

The role and accuracy of FOBT in the spinal cord injury (SCI) population are yet to be established. People with SCI are now living longer after their injury. Still, there remains a significant health disparity in overall life expectancy, with malignancy being a leading cause of morbidity and mortality in this group [8, 9]. Despite similar rates of CRC diagnosis at colonoscopy, people with SCI are less likely to undertake CRC screening with FOBT or surveillance colonoscopy [10–13]. Lower rates of colonoscopy are attributed to higher rates of inadequate bowel preparation, non-completion and significant peri-operative risks, such as autonomic dysreflexia [11, 12]. We postulated that the frequent use of rectal enemas, manual evacuation as well as chronic constipation and coexisting anorectal disorders, can lead to anal and rectal trauma, thereby leading to a higher rate of positive FOBT results.

Therefore, the aim of this study was to investigate the proportion of positive FOBTs among the SCI population compared to the general population as measured by NBSCP.

## Methods

This is a prospective study at a Quaternary referral centre in one of the two inpatient spinal injury units in the state of New South Wales, Australia. From June 2022 to June 2024, all persons aged 50–−74 years old admitted to hospital with an established SCI of more than 12 months were invited to participate in the study. People outside of the age criteria, with active per-rectal bleeding, haemorrhoids, rectal prolapses, anal fissures, per-vaginal bleeding, hematuria, spinal cord injury within 12 months, colonoscopy within 4 years, or had already completed FOBT as part of the NBCSP within 2 years were excluded. SCI classification were based on the American Spinal Injury Association (ASIA) and International Spinal Cord Society International Standards for Neurological Classification of Spinal Cord Injury. Recruited persons had two separate faecal samples taken on consecutive days and sent for immunochemical FOBT using prepared test kits. Faecal samples were taken when participants were stable and near expected date of discharge. No samples were taken when patients were critically ill or requiring intensive care admission. Those with at least one positive FOBT result were contacted by investigators and offered either an inpatient colonoscopy or outpatient follow-up with a colonoscopist at their local hospital. Those with a negative result were recommended to continue routine screening through the NBCSP. Information on demographics, previous colonoscopy results and bowel care regimens were collected. Anaemia was defined as < 125 g/L for males and < 115 g/L for females. Data were summarised using descriptive statistics with mean reported. Two-sample proportion test was used to assess the difference in rate of positive FOBT between this cohort and the population average in the NBCSP. A P-value less than 0.05 was considered significant. Informed consent was gained from all study participants. Ethics approval was granted by the Northern Sydney Local Health District Human Research and Ethics Committee (Reference Number 2020/ETH01533). All methods were performed in accordance with the relevant guidelines and regulations. Informed consent was obtained from all participants.

## Results

**Fig. 1:**
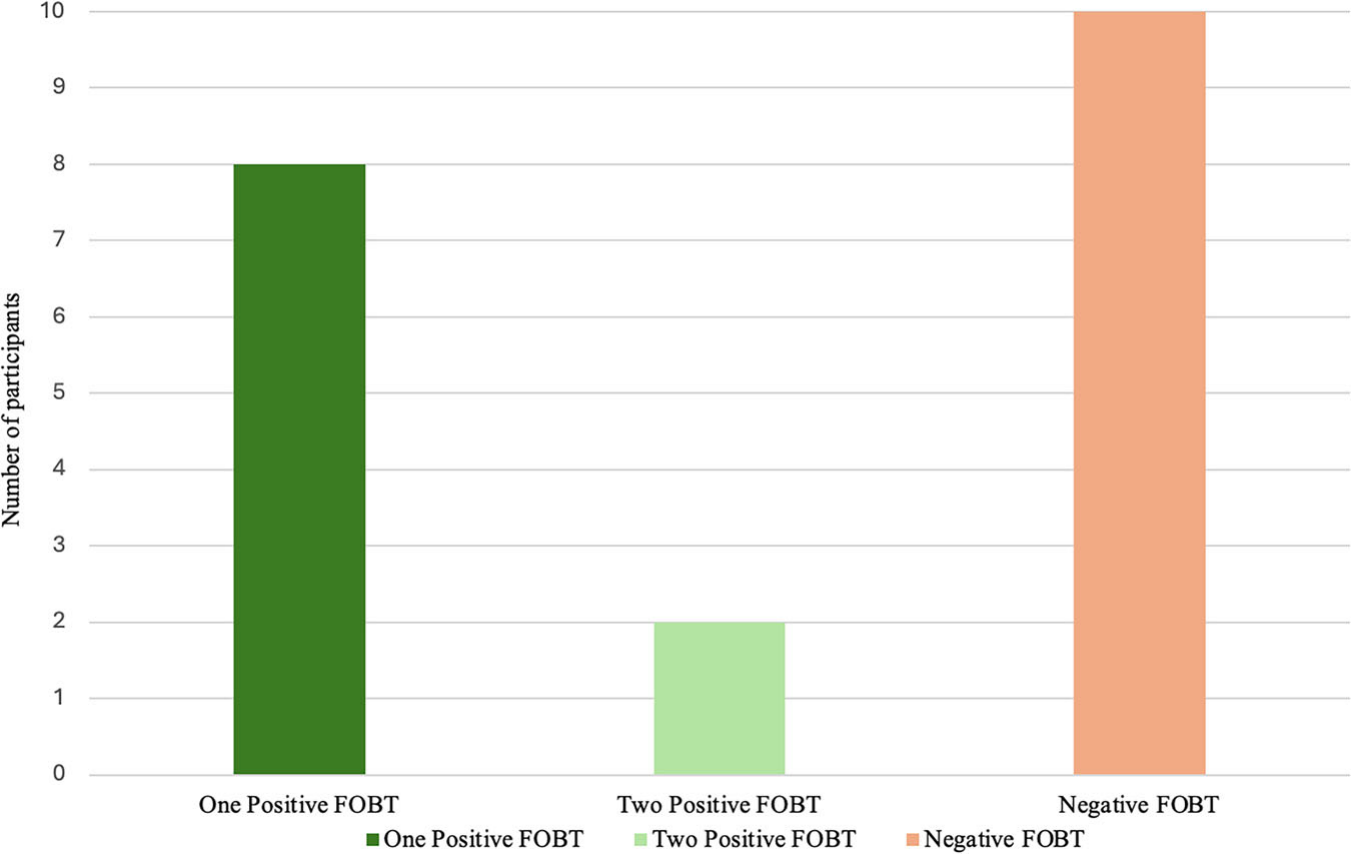
Bar graph showing the number of participants with either zero, one, or two positive FOBTs.

During the study period, 20 people were enrolled with a mean age of 60.6 ± 5.5 years-old. Demographic data and number of people returning positive FOBT are outlined in Table 1 and Fig. 1. There were 16 male and 4 female participants with a mean body mass index (BMI) of 25.6 ± 6.4 kg/m^2^. Admission indications included infection (*n* = 14/20, 70%), fall with fractures (*n* = 4/20, 20%), general decline (*n* = 1/20, 5%) and hypertension (*n* = 1/20, 5%). The median length of time lived with SCI was 31 years (IQR 4.5–37.5 years). There were an equal number of people with cervical and thoracolumbar level SCI. Ten participants were classified as ASIA Impairment Scale grade A, seven as grade B, two as grade C and one as grade D. Half of all participants returned at least one positive FOBT. Out of those who returned a positive FOBT, 90% (*n* = 9/10) had lived with SCI for more than 5 years and 50% (*n* = 5/10) were current or ex-smokers. Bowel care routines were individualised, with most incorporating oral osmotic laxatives (*n* = 15/20, 75%) and rectal techniques, including daily enemas (*n* = 14/20, 70%), digital stimulation (*n* = 13/20, 65%), or manual evacuation (*n* = 11/20, 55%). Only a minority used suppository (*n* = 1/20, 5%) or trans-anal irrigation methods (*n* = 2/20, 10%). Ninety percent (*n* = 9/10) of individuals who returned positive FOBTs reported using daily rectal techniques, such as enemas, manual evacuation, or digital stimulation, as part of their routine bowel care. At the time of faecal sample collection, three people were on aspirin and four people were on direct oral anticoagulation. Of the seven participants taking either antiplatelet or anticoagulant therapy, only two returned a positive FOBT. A high proportion of people were anaemic at time of testing (*n* = 17/20, 85.0%). A two-sample proportion test showed people with SCI had a significantly higher rate of positive FOBT (50%) compared to that of the general Australian population participating in the NBCSP (6.2%), *Z* = 8.11, *P* < 0.001 [14]. Almost one-third of FOBTs sent across all participants returned a positive result (*n* = 12/37, 32.4%). Of those who tested positive, 30% (*n* = 3/10) of participants agreed to proceed with colonoscopy. Of the people who underwent colonoscopy, two were incomplete due to poor bowel preparation, and one was normal. All persons who declined follow-up colonoscopy reported reasons of perceived difficulty with bowel preparation and procedural risks. Only 15% (*n* = 3/20) of participants had reported previously undergoing a colonoscopy, all of which were normal.

**Table 1:** Demographic characteristics of individuals with spinal cord injury (SCI): comparison between FOBT-positive and FOBT-negative groups.

	Positive FOBT	Negative FOBT	N (% total)	P value
Sex				
Male	8	8	16 (80%)	1
Female	2	2	4 (20%)	
Smoking Status				
Non-Smoker	5	7	12 (60%)	0.17
Current Smoker	2	2	4 (20%)	1
Ex-Smoker	3	1	4 (20%)	0.5
Spinal Cord Injury Level				
Cervical	7	3	10 (50%)	0.18
Thoracic or Lumbar	3	7	10 (50%)	
ASIA Impairment Scale Grade				
A	4	6	10 (50%)	0.32
B	5	2	7 (35%)	0.53
C	1	1	2 (10%)	1
D	0	1	1 (5%)	N/A
Length of Time Lived with Spinal Cord Injury				
<5 Years	2	3	5 (25%)	0.62
≥5 Years	9	6	15 (75%)	
Daily Bowel Care Routine				
Rectal Route				
Enema	9	5	14 (34.1%)	0.42
Digital Stimulation	6	7	13 (31.7%)	0.11
Manual Evacuation	5	6	11 (26.8%)	0.11
Trans-anal Irrigation	1	1	2 (4.9%)	1
Suppository	1	0	1 (2.4%)	N/A
Oral Medications				
Osmotic Laxative (Macrogol)	8	7	15 (60%)	0.14
Emollient Laxative (Docusate)	3	6	9 (36%)	0.27
Stimulant or Prokinetics	0	1	1 (4%)	N/A
Anticoagulation Use				
Yes	1	3	4 (20%)	0.58
No	9	7	16 (80%)	
Antiplatelet Use				
Yes	1	2	3 (15%)	1
No	9	8	17 (85%)	
Anaemia at Time of FOBT Sample				
Yes	7	10	17 (85%)	0.21
No	3	0	3 (15%)	

## Discussion

This is the first study that has evaluated the incidence of positive FOBTs in people with SCI. Our study demonstrates that people with SCI have a significantly higher positive FOBT rate of 50%, compared to 6.2% in the general population participating in the NBCSP [14]. Furthermore, people requiring anorectal instrumentation had a 90% positive FOBT rate.

False positive FOBTs are associated with demographic factors such as male sex, age ≥ 65, BMI ≥ 30, smoking and possibly antiplatelet use [15]. People with SCI are particularly prone to these risks owing to their restricted mobility, reduced activity levels, and reduced metabolic rate leading to a combination of sarcopenia and significantly higher rates of obesity and cardiovascular disease [16]. There is mixed evidence relating to the impact of antiplatelet and anticoagulation medication on the accuracy of FOBT and whether this increases the positive rate [17–19]. Only two of seven participants in our cohort who were on these medications returned a positive FOBT. Similarly, we found no relationship between anaemia and positive FOBT in our cohort of people with SCI. Anaemia was present in all persons who returned a negative FOBT (*n* = 10/10, 100%) yet this was present in only 70% (*n* = 7/10) of those who returned a positive FOBT. Whilst FOBT may be useful in screening for gastrointestinal bleeding and malignancy in those with iron deficiency anaemia, our evidence shows that this is not a reliable ancillary tool for people with SCI regardless of haemoglobin level [20–22].

Neurogenic bowel dysfunction affects up to 60–70% of people with SCI [23]. The loss of contracting abdominal musculature control, decreased colonic motility, and increased colonic and anal sphincter tone can cause significant issues with constipation, faecal impaction and incontinence [23, 24]. This exacerbates colonic and anorectal diseases such as anorectal prolapse, fissures and haemorrhoids resulting in frequent occult and macroscopic blood loss [15, 25, 26]. Faecal incontinence is often due to either a flaccid paralysis and loss of anal tone or from overflow incontinence. Many people with SCI require bowel management programs using a combination of rectal suppositories, enemas, trans-anal irrigation, digital stimulation, anal massage or manual evacuation to address and prevent complications associated with chronic constipation and incontinence [23, 27–29]. Bowel care with manual techniques or with devices using enema tips can lead to repetitive mechanical trauma to the anorectal mucosa with subsequent rectal bleeding [30, 31]. Most people in our cohort used at least one of these methods weekly and a significant majority of these (90%) returned a positive FOBT. Although clinicians have recognised that people with SCI may give a high false positive rate of FOBT, no quantitative data has been reported to support this observation until now [32]. Given the high rate of positive FOBT in our cohort of people with SCI, we highlight that this is not an appropriate method for CRC screening. More research is required to investigate whether primary screening with colonoscopy at set intervals may be a more safe and effective strategy for this population.

Despite a high rate of positive FOBT in our cohort, we had a low compliance rate with screening colonoscopy. Only one person proceeded to a complete colonoscopy, which did not show any abnormalities. One colonoscopy demonstrated multiple polyps and haemorrhoids, however despite several maneuvers did not achieve caecal intubation due to poor bowel preparation, redundant colon, bowel looping and a large body habitus. Another colonoscopy was incomplete due to inadequate bowel preparation and this person declined further investigation with a CT colonography. Most people in our cohort declined colonoscopy due to perceived difficulties with bowel preparation and procedural risk. Undergoing a colonoscopy is a burdensome task for people with SCI due to several challenging factors. Bowel preparation is technically difficult, prolonged, and resource-intensive, requiring carers for multiple bathroom transfers and frequent clean-up of stool [13, 33, 34]. Prolonged exposure to liquid stool poses a risk of peri-anal skin maceration and pressure injury development or contamination [33]. Reduced colonic motility and chronic constipation result in higher rates of inadequate bowel preparation, lower colonoscopy completion rates and an increased need for repeat procedures [11, 33]. Furthermore, people with SCI have high peri-operative morbidity and mortality due to their susceptibility to autonomic dysreflexia, respiratory failure and cardiovascular disease [11, 35]. Studies have shown an equivalent CRC risk but lower screening, colonoscopy surveillance and polyp detection rates between the general and SCI populations [11, 12, 34]. Whilst there is no universally established optimal bowel preparation protocol for people with SCI, an admission for inpatient extended bowel preparation is often required [11, 33]. All people with SCI at our institution requesting colonoscopy were referred for pre-operative anaesthetic clinic assessment with inpatient admission three days before the procedure for extended bowel preparation. With adequate procedure preparation, people with SCI can still have similar safety outcomes and adenoma detection rates as the general population [13]. Due to the multiple difficulties people with SCI face in the peri-procedural period, it is prudent that healthcare systems develop more streamlined systems for this population to encourage better compliance and completion of CRC screening [11].

The strength of our study lies in the prospective enrolment of people with SCI, with inclusion criteria mirroring that of the NBCSP. Our data has real-world applicability with new evidence to support bypassing the use of FOBT for CRC screening in people with SCI. Given the high positive FOBT rate (50%), participation in the NBCSP by people with SCI may lead to unnecessary and repetitive colonoscopy procedures with avoidable peri-procedural risks. Our study is limited by the low patient sample size and colonoscopy uptake throughout the 2-years of recruitment. Since only a minority of patients elected to proceed with colonoscopy, it is difficult to ascertain whether those who tested positive on FOBT have an underlying malignant pathology contributing to positive results. This highlights the multi-faceted challenges faced by people with SCI participating in CRC screening. Barriers to screening include healthcare accessibility, transport organisation, psychological fear, environmental barriers, and reluctance to proceed with colonoscopy due to high emotional and physical demands [36, 37]. The financial implications of appropriate CRC screening in the SCI population are important and two-fold. Firstly, inpatient bowel preparation often requires an extended hospital admission occupying valuable time and staffing resources. Secondly, if screening is not adequately performed then progression to colorectal cancer will lead to further excess healthcare costs in this population [38]. Due to the difficulty with colonoscopy in this population, less invasive screening strategies without the requirement for prolonged admissions such as using FDG-PET scans could be considered [39]. For those with incomplete colonoscopy, CT colonography with a sensitivity of 88.8% and specificity of 75.4% for colonic neoplasia should be offered. Further studies will also need to evaluate optimal bowel preparation protocols and how healthcare services may encourage better follow-up compliance with CRC in those with SCI.

## Conclusion

To date, there has been a lack of studies investigating the accuracy of FOBT in people with SCI. In this study, 50% of people with SCI returned a positive FOBT, much higher than rates seen in the general Australian population of 6.2%.

The high positive rate of FOBT in the SCI population suggests that it may not be an effective screening tool. Instead, alternative methods, such as universal screening colonoscopy, may be warranted. Follow-up colonoscopy rates were poor and compliance may be improved by more streamlined inpatient bowel preparation protocols. Higher rates of positive FOBT may be due to complications of chronic constipation, and colonic and anorectal trauma.

## Data Availability

The data that support the findings of this study are available on request from the corresponding author. The data are not publicly available due to privacy or ethical restrictions.
